# Cost-effectiveness of hospice-based palliative care in Kazakhstan

**DOI:** 10.1093/eurpub/ckac131.241

**Published:** 2022-10-25

**Authors:** I Salikhanov, B Crape, S Wieser, M Katapodi

**Affiliations:** 1 Medical Faculty, University of Basel, Basel, Switzerland; 2 School of Medicine, Nazarbayev University, Nur-Sultan, Kazakhstan; 3 ZHAW School of Management and Law, Zurich University of Applied Sciences, Zurich, Switzerland

## Abstract

**Background:**

According to the Quality of Death Index, Kazakhstan ranks 50th out of 80 countries assessed. Currently, some form of inpatient end-of life care in Kazakhstan is provided by only 9 hospices, palliative care units and mobile teams. In 2020, the total number of palliative care beds did not exceed 980 across the country, while around 135,000 patients need palliative care at any given time.

Objective: To assess the effectiveness and cost-effectiveness of hospice-based palliative care for cancer patients compared to usual hospital care in cancer centers across Kazakhstan from societal perspective.

**Methods:**

A total of 182 family caregivers were recruited, 104 in a hospice group and 78 in a control (palliative units). Patients’ state of health and family caregivers’ burden been measured using Palliative Outcome Scale (POS) and Zarit Burden Inventory (ZBI) on 14th day of the inpatient palliative care. Direct and indirect medical costs as well as family caregivers’ out-of-pocket expenses associated with the care has been collected. The cost-effectiveness analysis was conducted by calculating the difference between mean cost of treatment, including OOPs, over 14 days of treatment. Uncertainty around the cost-effectiveness estimates was explored by generating 10,000 resamples using bootstrapping.

**Results:**

At 14 days, patients’ mean quality of life was 2,4 points better (95% CI: 0,06 - 4,9) in the hospice group compared to the control. Family caregiver burden was 4,6 points better in the hospice group (95% CI: -0,26 - 9.3). Mean treatment costs, including direct medical costs and out-of-pocket expenditures over 14 days were $31 lower for the hospice group (95% CI: $29 - $32). There was a significant correlation between the total cost of treatment and patients’ quality of life (rxy = 0,58; p < 0,01). The cost-effectiveness plane graphically represents 10,000 replications, 85% of them showed that hospice care has better outcomes and lower costs than the control group.

**Key messages:**

• Hospice-based palliative care is cost-effective compared to the care provided in palliative units of cancer centers.

• There is a significant correlation between patients’ quality of life and family caregivers’ burden.

